# Anemia among pregnant women in internally displaced camps in Mogadishu, Somalia: a cross-sectional study on prevalence, severity and associated risk factors

**DOI:** 10.1186/s12884-021-04269-4

**Published:** 2021-12-14

**Authors:** Ramla Hussein Ahmed, Asha Abdirahman Yussuf, Asma Abdikarin Ali, Sowdo Nuur Iyow, Maryan Abdulahi, Lul Mohamud Mohamed, Mohamed Hayir Tahlil Mohamud

**Affiliations:** 1 Faculty of Medicine & Surgery, Jazeera University, Mogadishu, Somalia; 2 Paediatric Department, Jazeera University Hospital, Mogadishu, Somalia; 3 Obstetrics and Gynaecology Department, Dr Sumait Hospital, Mogadishu, Somalia; 4 Faculty of Medicine & Health Sciences, SIMAD University, Mogadishu, Somalia; 5 Research Unit, Jazeera University, Mogadishu, Somalia

**Keywords:** Anemia, Pregnant women, IDP, Camps, Mogadishu, Jazeera University

## Abstract

**Background:**

Anemia in pregnancy is a serious global public health problem in most developing countries and a major cause of maternal morbidity and mortality. Somalia which already had very high maternal mortality ratio of 829 per 100,000 live births, pregnant women in internally displaced camps (IDPs) remain at most exposed. The aim of the study was to determine the prevalence, severity and associated risk factors of anemia among pregnant women in internally displaced camps in Mogadishu, Somalia.

**Methods:**

A community based cross-sectional study was conducted among 383 households in the most IDP settled districts in Mogadishu. Every pregnant mother in these sampled households who was voluntarily consented was targeted. A sample of blood was also taken by pricking the fingertip and inserted into hemoglobin meter. Those with Hb < 11 g/dl from hemoglobin meter had been taken another sample of 3 cc blood and put into EDTA tube for CBC analysis to identify the type of anemia. Data on risk factors were collected using structured pretested questionnaire via an interview. Collected data was coded and entered in SPSS- Version 22 for analysis. Descriptive analysis, bivariate chi-square and multivariate logistic regression were done.

**Results:**

The overall prevalence of anemia among study participants was 44.4% (95%CI: 39.5-49.3%), where severe and moderate anemia were 11.8 and 47.0% respectively. In addition all anaemic cases were microcytic hypochromic anemia. Young maternal age, low Family income, fewer/zero parity, being at third or second trimesters, lack of ANC attendance during pregnancy, lack of iron supplementation during pregnancy, taking tea immediately after meal during pregnancy, lower/zero frequency of daily meat and vegetables consumption during pregnancy were associated risk factors of anemia.

**Conclusion:**

The anemia prevalence from this study was severe public health problem. Several factors were found to be associated with anemia during pregnancy. Measures has to be taken to curb the problem by including them mass iron supplementation and health education towards identified risk factors.

**Supplementary Information:**

The online version contains supplementary material available at 10.1186/s12884-021-04269-4.

## Background

Anemia is a public health problem that affects 1.62 billion people worldwide, which corresponds to 24.8% of the world population [1]. It is characterized by reductions in hemoglobin concentration, red-cell count, or packed-cell volume.

Anemia is a common health problem for women and children of developing countries [2, 3]. (WHO) has defined anemia during pregnancy as hemoglobin (Hb) concentration less than 11 g per deciliter (g/dl) [4]. Pregnant women in developing countries are prone to anemia due to low socioeconomic conditions. The poor nutritional intake, repeated infections, frequent pregnancies and low health-seeking behaviors are associated with anemia [2, 5]. It is estimated that 40% of pregnant women globally are anemic, with iron deficiency anemia being the most common type [6]. Annually, nearly 510,000 maternal deaths occur worldwide, associated with childbirth or early postpartum complications. Approximately 20% of this maternal death is caused by anemia; the majority of this is taking place in developing countries [7]. Of the 41.8% of pregnant women it affects globally, the highest prevalence are in Africa (57.1%) which corresponds to 17.2 million [5].

Anemia in pregnancy is associated with increased risk of preterm birth and low birth weight babies [8–11]. Preterm and LBW are still the leading causes of neonatal deaths in developing countries [12]. It has also been associated with increased risk of intrauterine deaths (IUFD), low APGAR score at 5 min, and intrauterine growth restriction (IUGR) which is a risk for stunting among children of less than 2 years [8, 9, 13].

Pregnant women in refugees and internally displaced camps are the most vulnerable once, not only for the morbidity and mortality of anemia alone but many other diseases and conditions. A report from Dadab refugee camp in Kenya shown that all maternal deaths reported in 2008, anemia accounted 55% of them [14]. The aim of this study was to determine the prevalence, severity and associated risk factors of anemia among pregnant women in internally displaced camps in Mogadishu, Somalia.

## Method and materials

### Study design

A community based cross-sectional study was carried out from February to July 2020 to determine the prevalence, type, severity and risk factors of anemia among pregnant women living in IDP camps in Mogadishu-Somalia.

### Study population and setting

Recurrent droughts and conflicts in many parts of the country forced many people to displace internally. Most displaced settlements locate in the outskirts of Mogadishu, especially in the districts of Deynile, Kaxda and Hodan which were all under this study. As the data from the (Internal Displacement Profiling, 2016) showed, the IDP population comprises 33% of all households (or 151,861 individuals) in Daynile district, followed by Kaxda 19% of all households (or 88,091 individuals) and Hodan 17% of all households (or 83,374 individuals).

### Measurement of variables

#### Outcome variable

The outcome variable of this study is anemia. Anemia in pregnancy is defined as Hb status less than 11 g/dl. On the bases of its severity, anemia was categorized as severe anemia (< 7.0 g/dl), Moderate anemia (7.0-9.9 g/dl) and Mild anemia (10.0-10.9 g/dl) [15].

### Independent variables

#### Sociodemographic variables

Included women age (14-21 years, 22-29 years, 30-37 years and 38-45 years), educational status (illiterate, informal education, primary education and secondary & above education), occupation (employed and Housewife) and daily family income (≤ $1 dollar, $1-3 dollars and > $3 dollars).

#### Obstetric variables

Included parity (0, 1-3 and > 3), pregnancy trimester (First, second and third), birth space (< 2 years and ≥ 2 years), ANC visit during current pregnancy (never visited, 1-3 visits and > 3 visits) and Iron supplementation during current pregnancy (Yes and No).

#### Dietary variables

Included consumption of tea immediately after meal (Yes and No), frequency of daily meat consumption (None, one time, two times and three times) and frequency of daily vegetable consumption (None, one time, two times and three times).

### Sample size determination and sampling procedure

**Fig. 1:**
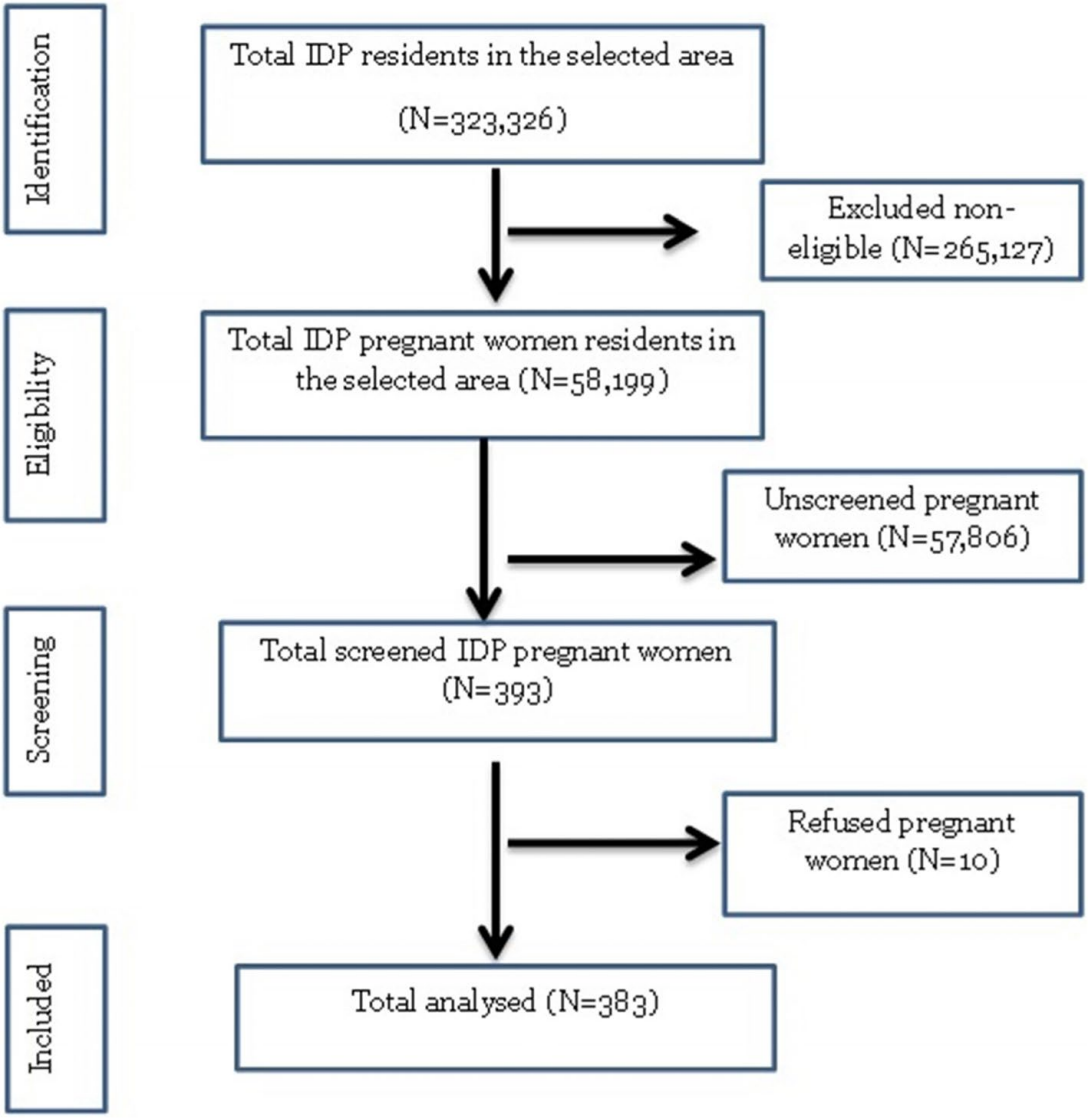
Study flow chart

The sample size was calculated using single proportion formula (*n* = [(zα/2)^2^ ×  (1-p)]/d^2^) with the level of confidence of a 95, 5% margin of error, 36.1% prevalence of anemia in pregnant women from study in South Sudanese Refugees in Pugnido, Western Ethiopia refugee camps [1] and a 10% nonresponse rate which yielded 393. Out of the 17 districts in Mogadishu, three periphery districts where most IDP settlements locate have been selected for this study. These districts were Deynile where IDP settlements comprise (33%), Kaxda (19%) and Hodan (17%) (Internal Displacement Profiling, 2016). Based on this proportion, the sample size was allocated into 27% (107 households) to Kaxda, 47% (185 households) into Deynile and 26% (101 households) into Hodan. Households in each of these sampled districts were selected using systematic random sampling technique, where every 3rd household were chosen (Fig. 1).

### Sample collection and processing procedure

Pregnancy of the mother was confirmed through LNMP method if she was in the first trimester or abdominal examination if in second and third trimester. For the confirmed consented pregnant women information on Socio-demographic, obstetric and dietary intake was collected using a structured questionnaire. The questionnaire was developed into English then translated to Somalia then back to English. A sample of blood was also taken by pricking the fingertip after use of septic technique, then test strip was inserted into the device and the results were then recorded. Those with hemoglobin level less than 11 g/dl from hemoglobin meter results had been taken another sample of 3 cc blood through venepuncture and put into EDTA tube for CBC analysis to identify the type of anemia. Throughout the sample there was no difference in the results from the hemoglobin meter and CBC analysis. Every subject’s sample and her questionnaire were labelled into unique identification number. After the test, pregnant women who were mild to moderate anemia were given iron supplement (Two times daily oral iron and folic acid supplementation with 60 mg of elemental iron and 400 μg folic acid for a period of 3 months with follow up) and health education. Those with severe anemia were given health education and facilitated to come into Banadir Hospital immediately via an ambulance for further investigations and treatments. Finally, Participants whose CBC analysis was < 11 g/dl were regarded to be anemic.

### Data analysis and processing

The collected data was coded and entered in SPSS- Version 22 for analysis. Descriptive analysis such as mean, standard deviation, frequency and percentages was performed to summarize the socio demographic, obstetric, and nutrition related data.

A bivariate chi-square analysis was used to determine the significance of the associations between the variables. Variables with a *p*-value ≤ 0.2 were taken into multivariate level. Multivariable logistic regression analysis was performed using the Backward stepwise regression method to control potential confounding variables. Furthermore, correlation between the explanatory variables was assessed to test multi-collinearity. VIF > 5 were considered the existence of Multicollinearity between the explanatory variables. Finally, the strength of association was measured by adjusted odds ratios with a 95% confidence interval (CI) for exposure variables and the outcome variable (Anemia). *P*-value< 0.05 was regarded statistically significant in all conditions of the analysis.

## Results

Data from 383 study participants were included in the analysis out of 393 calculated samples, with 97.5% response rate.

### Socio demographic and obstetric characteristics

As shown in Table 1 the Mean ± SD age of subjects was 26.10 ± 6.54 years. Nearly two-third of the participants (243, 63.4%) were housewives. Majority of the participants (279, 72.8%) were illiterate and only two participants (0.5%) completed secondary or higher education. The Mean ± SD of participants’ daily income was 2.26 ± 1.09 USD per day with majority (308, 80.4%) had 1-3 dollars per day. In parity, 60.1% (230) of the women had more than three children with the Mean ± SD parity of 4.7 ± 3.23 children. In addition about two-third of the subjects had birth space of less than 2 years from the current pregnancy (229, 66.8%). Although majority of the subjects were in their Second and third trimesters (38.4 and 36.0%) respectively, more than half (220, 57.4%) never visited ANC during the current pregnancy. That leads only 38.1% to take Iron supplements.

**Table 1:** Socio-demographic and Obstetric characteristics of pregnant women in IDPs of Mogadishu, 2020

	Frequency	Percentage%
**Age in years**		
14-21 years	114	29.8
22-29 years	133	34.7
30-37 years	119	31.1
38-45 years	17	4.4
Mean ± SD=	26.10 ± 6.54 years	
**Occupation**		
Employed	140	36.6
Housewife	243	63.4
**Level of education**		
Illiterate	279	72.8
Informal	87	22.7
Primary	15	3.9
Secondary and above	2	0.5
**Income per day in dollars**		
≤ $1	41	10.7
$1-3	308	80.4
> $3	34	8.9
Mean ± SD=	2.26 ± 1.09 UD	
**PARITY**		
0	40	10.4
1-3	113	29.5
> 3	230	60.1
Mean ± SD=	4.7 ± 3.23 children	
**Pregnant Trimester**		
First	98	25.6
Second	147	38.4
Third	138	36.0
**Birth space in years (*N*** **= 343)**		
< 2 years	229	66.8
≥ 2 years	114	33.2
Mean ± SD=	1.35 ± 0.589 years	
**ANC Visits**		
Never visited	220	57.4
1-3 Visits	149	38.9
> 3 Visits	14	3.7
**Iron Supplement**		
Yes	146	38.1
no	237	61.9

### Prevalence, severity and type of Anemia among study participants

The overall prevalence of anemia among pregnant women living in Internally Displaced camps of Mogadishu, Somalia was found to be 44.4%(170/383) with the mean (±SD) Hb concentration of 10.37 (±1.93) g/dL. Among them, severe anemia was 11.8% (20/170), moderate anemia was 47.0% (80/170) and mild anemia was 41.2% (70/170). All anaemic pregnant women had microcytic hypochromic anemia (170/170). The mean (±SD) of anemia from Hb meter was 9.019(±1.0772) g/dL while the mean (±SD) of anemia from CBC was 9.315 (±1.2795) g/dL. The overall mean (±SD) Hb concentration of all subjects was 10.37 (±1.93) g/dL. (Table 2).

**Table 2:** Prevalence, Severity and Type of Anaemia among pregnant women in included in the study in IDPs of Mogadishu, 2020

		SEVERITY (%)		
Anemic status	FREQUENCY (%)	Mild	Moderate	Severe
Anaemia	170(44.4%)	70(41.2%)	80(47.0%)	20(11.8%)
Non-anaemia	213(55.6%)			

### Multivariate logistic regression

Age, Family income and parity were all had a negative association with anemia, that is an increase on age, daily family income and parity by a unit would likely to decrease the risk of being anemia by a factor of 0.967, 0.717 and 0.878 respectively (*p*-value< 0.05).

Pregnant women in the third and second trimesters were 4.48 and 2.66 times respectively more likely to be anaemic than those with first trimester [(AOR: 4.483; 95% CI: 22.384-8.431; *P*-value = 0.001); (AOR: 2.666; 95% CI: 1.466-4.848; *P*-value = 0.001)]. Pregnant women who never visited ANC during pregnancy were found seven times more likely to be anaemic compared to those who had more than three visits (AOR: 6.707; 95%CI; 1.390-32.352; *P*-value = 0.018). Pregnant women who did not take iron supplement during their pregnancy were two times more likely to be anaemic compared than those who took it (AOR: 2.166; 95%CI: 1.410-3.327; *P*-value 0.001). Pregnant women who took tea immediately after meal were 3.4 times more likely to be anaemic than those who had no (AOR: 3.430; 95% CI: 2.004-5.873; *P*-value = 0.001). A decrease in the frequency of daily meat intake during pregnancy were found to be associated with increased risk of anemia, women who had None, once and twice a day were 19.595, 15.633 and 12.554 respectively more risk to be anaemic than those who had three times per day [(AOR: 19.595;95% CI: 2.264-169.606; *P*-value = 0.007); (AOR: 15.633;95% CI: 1.992-122.700; *P*-value = 0.001); (AOR: 12.554;95% CI: 1.536-102.633; *P*-value = 0.018)]. Similarly, A decrease in the frequency of daily vegetable consumption during pregnancy were found to be associated with increased risk of anemia, In addition, that is women who never took green vegetables or took once in a day during their pregnancy were twelve and five times more likely to be anaemic respectively than those who took more than three times per day during their pregnancies [(AOR: 11.976; 95% CI: 4.569-31.391; *P*-value = 0.001); (AOR: 5.475; 95% CI: 2.413–12.423; *P*-value = 0.001)]. **(**Table 3**).**

**Table 3:** Multivariate logistic regression results of factors associated Anemia among pregnant women in included in the study in IDPs of Mogadishu, 2020

Variable	AOR for 95% CI	Sign.
**Age**	0.967(0.937-0.998)	0.038
**Daily income**	0.717(0.577-.891)	0.003
**Parity**	0.878(0.810-0.951)	0.001
**Pregnant Trimester**		
Third Trimester	4.483(2.384-8.431)	0.001
Second Trimester	2.666(1.466-4.848)	0.001
First Trimester	1	
**Number of ANC visit during current pregnancy**		
None	6.707(1.390-32.352)	0.018
1-3 times	3.539(0.727-17.229)	0.118
> 3 times	1	
**Iron supplement**		
No	2.166(1.410-3.327)	0.001
Yes	1	
**intake of meal with Tea**		
Yes	3.430(2.004-5.873)	0.001
No	1	
**Meat intake per day**		
None	19.595(2.264-169.606)	0.007
One time	15.633 (1.992-122.700)	0.009
Two time	12.554 (1.536-102.633)	0.018
Three time	1	
**Green vegetable intake per day**		
None	11.976 (4.569-31.391)	0.001
One time	5.475 (2.413-12.423)	0.001
Two time	1.964 (0.847-4.554)	0.116
Three time	1	

*KEY*: *β* Beta, *AOR* Adjusted Odd Ratio, *CI* Confidence Interval = 95%, *P*-value < 0.05 is significant

## Discussion

The overall prevalence of anemia among the subjects of this study was found to be 44.4% (170/383) with the mean (±SD) Hb concentration of 10.37 (±1.93) g/dL.

Based on WHO cut off values, anemia prevalence in this study is regarded as severe public health problem [16]. The overall prevalence of anemia among all pregnant women in Somalia was declining slowly from 50.0 to 46.8% between 1990 and 2016 [17]. The anemia prevalence of this study, 44.4% was higher than the findings in a study conducted in South Sudanese refugees in Pugnido at western Ethiopia (36.1%) [1], Southeast Ethiopia 27.9% [7], Palestinian refugees in Occupied Palestinian Territory, 38.6% [18], South Sudanese refugee living in refugee camps of Uganda (36.3%) [19] and Malaysia (35.0%) [20]. This difference might be due to timing and methodological difference, for example the studies conducted at South Sudanese refugees, Pugnido, western Ethiopia and Palestinian refugees in Occupied Palestinian Territory was facility based conducted at 2015 and 2006 respectively. Other study conducted at South Sudanese refugee in New Caseload of Uganda [19] was a large sample size study done at 2007 while the Southeast Ethiopia study [7] was facility based for residential pregnant women. Same was the Malaysian study [20] although it was targeted residential population in additional to these differences, displaced people in these areas may have better aid and other facility access than IDPs in Somalia.

This prevalence was slightly lower than 2016 World Bank report on “prevalence of anemia among pregnant women in Somalia” (46.8%) [21]. This is may be due to that pregnant women in IDPs are very vulnerable groups with no access to balanced diet, poor access to health and low knowledge. It was also lower than other studies conducted in Dadab refugee camps (75%) [22] and Saharawi refugees in Algeria (55.1%) [23]. This might be due to timing; for example, reports from Dadab refugee camps was conducted in 2008 while the study on Saharawi refugees in Algeria [23] was large sample sized study conducted at Refugee Camps, Tindouf, Algeria.

Moderate anemia was the common form of anemia in this study with 47% of the pregnant women. Moderate anemia was also reported as the major form of anemia among pregnant women from studies conducted in Saharawi refugees in Algeria, (25.5%) [23], Eastern Sudan (52.4%), Wolayta Sodo Town, Southern Ethiopia (60%) [24], Karnataka, India (50.4%) [25] and West Algeria (49.5%) [26]. There were many other studies where mild type of anemia was the predominant form, these were included a study conducted in 2015 at South Sudanese refugee living in refugee camps of Uganda, (89.2%) [1], Southeast Ethiopia study, (55%) [7], Palestinian refugees in Occupied Palestinian Territory, (35%) [18], South Sudanese refugee in New Caseload of Uganda (86.2%) [19], Tikur Anbessa, Addis Ababa Ethiopia (80.9%) [27] and Kakamega, Eastern Sudan (52.4%) [28]. The possible sources of inconsistency with the study done in Sudanese refugee living in refugee camps of Uganda might be time it was conducted, 2015 and since it was facility based. The other study in Southeast Ethiopia study was small sample sized facility based that did not include IDP or refugee. Studies on Palestinian refugees in Occupied Palestinian Territory and Kakamega, Eastern Sudan used different cut-off points for mild (9-10.9) and moderate (7-8.9) while Tikur Anbessa, Addis Ababa Ethiopia study was facility based on non IDP/refugee pregnant women that also excluded any pregnant women who were recently transfused, or had chronic medical diseases, diagnosed hemoglobinopathies, or who had early bleeding or antepartum hemorrhage.

Considering the morphological classification of anemia, all the patients had microcytic hypochromic anemia (170/170). This is in agreement with a report from Turkey [29] and West Algeria [26]. This is also in line with WHO report [16] that the commonest cause of anemia in pregnancy is nutritional problem especially iron deficiency.

Socio-demographic factors can play a big role in determining anemia among pregnant woman. In this study only maternal age and daily family income were significantly associated with Hb status. Anemia was significantly more prevalent among young pregnant women (AOR: 0.967; 95% CI: 0.937-0.9981). Similarly pregnancy anemia was 28.3% more prevalent among women with family income ≤$1 per day than those >$1 per day (AOR: 0.717; 95% CI: 0.577-0.891). Age and family were documented as important risk factors of anemia among pregnant women in different literatures [1, 29]. Poor income leads to limited access to nutritious diets and is associated with poor eating habits that might lead to anemia. On other hand, If an adolescent girl becomes pregnant, the mother and foetus will compete for nutrients to support their rapid growth which in turn increases her vulnerability for anemia [30]. Insecurity in the area of IDP camps, aid shortage and mismanagement of aid distribution creates families not only to rely on aid donation. Hence financial constraints are challenging to access and afford nutritious foods [31]. Other socio-demographic factors like educational level and occupation were found as insignificant predictors of anemia in this study but were significant in other studies [32, 33].

Many obstetric characteristics play an important role in determining Hb status through increasing requirement for iron or depletion of its storage. In this study anemia were 4.48 (AOR) and 2.66 (AOR) more prevalent in pregnant woman with third and second trimesters respectively compared to those in the first trimesters. Because the requirements for absorbed iron increase from 0.8 mg/day in the first trimester to 7.5 mg/day in the third trimester [34, 35]. This finding was in line with WHO report [36]. Parity was found to have a negative association with anemia (AOR = 0.878), that is anemia were more prevalent among nulliparous women (or women with fewer children) compared to multiparous. This is may be that less parity women are less expert in pregnancy related complications with addition; more 95% of the subjects in the study had no basic education and all were poor IDPs. They may feel more difficult in the morning sickness that may lead to refuse the available food with no other option of choice. In contrast the higher party women may have higher experience in pregnancy, morning sickness, anemia and its management. This finding was consistence in studies in Ethiopia [37], Ghana [38] and Turkey [29].

According to the WHO [36], an increase in ANC attendance during pregnancy may decrease the risk of maternal mortality and morbidity including pregnancy anemia. In this study, pregnant women who had zero ANC visits or 1-3 ANC visits were 6.7 and 3.5 times more likely to be anemic respectively compared those who had more than three visits. This is consistence with many literatures that shown an increase in ANC attendance during pregnancy is associated with a decrease of risk of pregnancy anemia [39, 40].

Inadequate consumption and improper dietary habit with respect to the necessary micronutrient during pregnancy can increase the risk of developing anemia. The result of this study shown that anemia was 2.266 more prevalent in pregnant women who did not take iron supplements during pregnancy than those who had (AOR = 2.166; 95% CI: 1.410–3.327). this is because Requirements for absorbed iron increase in an average of ~ 4.4 mg/day in the entire gestation period [34, 35]. In the current study, tea intake immediately after meal had been associated with increased risk of 3.4 times to be anaemic (AOR: 3.430; 95% CI: 2.004–5.873). This was concordant with the findings from Sudanese Refugees in Pugnido at Western Ethiopia [1], West Arsi Zone in Ethiopia [41] and Fayoum Governorate in Egypt [42]. Frequency of daily meat and vegetable intake were found to be positive predictors of anemia, where pregnant women with higher frequency of meat consumption or higher frequency of vegetable consumption had lower risk of anemia (*P*-value< 0.05). Same result was documented in study conducted in Bangladesh [43], Nepal [43]. This is because meat and vegetables are sources of iron [43, 44].

### Limitations of the study

This study showed the burden and risk factors of anemia among the most vulnerable population groups, pregnant mothers, in IDPs camps where there is scarcity of literature. It may also help policymakers to develop strategies to prevent and reduce this burden. However; the study has some limitations that need to be taken into account when interpreting the study results. First, the study was done only three internally displaced camps in Mogadishu; therefore we cannot generalize the finding from this study to all internally displaced pregnant women in Somalia. Second, the study did not include Malaria and HIV tests or even stool examinations for parasitic tests which would be possible explanations for some findings. Even the history of fever for malaria and loose stool for GI parasites was not taken. Third, no physical examination was done. Forth, the CBC analysis was made only for the patients with for Hb meter  <11 g/dl.

## Conclusion

Prevalence of anemia from this study was a regarded as severe public health problem based on WHO cut off values. The severity of the anemia was moderate form. All anaemic pregnant women had microcytic hypochromic anemia. maternal age, daily family income, parity, pregnancy trimesters, frequency of ANC attendance, iron supplementation, tea intake immediately after meal, frequencies of meat and vegetable intake were associated risk factors of anemia among pregnant women in IDP camps in Mogadishu-Somalia. Awareness creation on the consequences of anemia during pregnancy should be given to IDP women in child bearing age in general and pregnant women in particular. Measures has also be taken to curb the problem by including them mass iron supplementation and health education towards identified risk factors.

## Supplementary Information

**Additional file 1.**

## Data Availability

The datasets used and analysed during the current study are available from the corresponding author on reasonable request.

## Abbreviations

AOR: Adjusted Odds Ratio
ANC: Antenatal care attendance
CBC: Complete Blood Count
CI: Confidence Interval
GI: Gastrointestinal
Hb: Hemoglobin
IDP: Internally Displaced People
LBW: Low Birth Weight
SD: Standard Deviation
