# Supplementary material for: Anemia among pregnant women in internally displaced camps in Mogadishu, Somalia: a cross-sectional study on prevalence, severity and associated risk factors
Source: BMC Pregnancy Childbirth. 2021 Dec 14;21:832. doi: 10.1186/s12884-021-04269-4 (PMC8670163; doi:10.1186/s12884-021-04269-4)
Supplement: Supplementary file 1 — Additional file 1. [file 12884_2021_4269_MOESM1_ESM.docx]

**QUESTIONNAIRE IN ENGLISH**

Dear Madam, kindly help to fill this questionnaire guide. The purpose of this guide is to collect data about risk factors of anemia among pregnant women in IDPS at Kaxda, Deynile and Hodan districts in Banadir region.

Please tick or fill in the blank spaces in the table of brackets against any alternative’s response items provided for each question. Where possible you can also write in the blank spaces provided for some of the open questions.

**PART ONE: - SOCIO - DEMOGRAPHIC ASSESSMENT:**

1. How old are you?

-----------------------years

1. Occupation
2. Employed
3. Unemployed
4. Education of the respondent
5. Illiterate
6. Informal
7. primary
8. Secondary and above
9. How much is your Daily income (USD)?

--------------------------------------

**PART TWO: - OBESTATRIC FACTORS:**

1. How many children do have?

------------------------------------------------

1. Pregnancy trimester
2. First trimester
3. Second trimester
4. Third trimester
5. What is the age interval between your children?

-----------------------------------------------------------

1. ANCvisits.
2. Never
3. 1-3 times
4. > 3 times
5. Do you take iron supplements During pregnancy?

a) Yes

b) No

**PART THREE: - DIETARY FACTORS:**

1. Do you take tea immediately with meal during this pregnancy?
2. Yes
3. No
4. How many times do eat meat in a day during this pregnancy?

------------------------------------------

1. How often do you eat green vegetables in a day during this pregnancy?

-------------------------------------------

1. Hemoglobin level (Hb Meter)

------------------------------------------

1. Hemoglobin level (CBC)

------------------------------------------

5. Type of Anemia (from CBC analysis)

1. Microcytic hypochromic anaemia
2. Macrocytic hyperchromic anaemia
3. Normocytic normochromic anaemia
4. Normal
